# Recent advances in vaccine and immunotherapy for COVID-19

**DOI:** 10.1080/21645515.2020.1825896

**Published:** 2020-11-06

**Authors:** Ali A. Rabaan, Shamsah H. Al-Ahmed, Ranjit Sah, Jaffar. A. Al-Tawfiq, Ayman M. Al-Qaaneh, Lamiaa H. Al-Jamea, Alexander Woodman, Manaf Al-Qahtani, Shafiul Haque, Harapan Harapan, D. Katterine Bonilla-Aldana, Pavan Kumar, Kuldeep Dhama, Alfonso J. Rodriguez-Morales

**Affiliations:** a Molecular Diagnostic Laboratory, Johns Hopkins Aramco Healthcare, Dhahran, Saudi Arabia; b Specialty Paediatric Medicine, Qatif Central Hospital, Qatif, Saudi Arabia; c Department of Microbiology, Tribhuvan University Teaching Hospital, Institute of Medicine, Kathmandu, Nepal; d Specialty Internal Medicine, Johns Hopkins Aramco Healthcare, Dhahran, Saudi Arabia; e Department of Medicine, Indiana University School of Medicine, Indianapolis, IN, USA; f Department of Medicine, Johns Hopkins University School of Medicine, Baltimore, MD, USA; g Department of Genetic Research, Institute for Research and Medical Consultations (IRMC), Imam Abdulrahman Bin Faisal University, Dammam, Saudi Arabia; h Clinical Pharmacy Services Division, Pharmacy Services Department, Johns Hopkins Aramco Healthcare, Dhahran, Saudi Arabia; i Clinical Laboratory Sciences, Prince Sultan Military College of Health Sciences, Dhahran, Saudi Arabia; j Department of Medicine, Royal Medical Services, Bahrain Defence Force Hospital, Manamah, Bahrain; k Department of Medicine, Royal College of Surgeons in Ireland-Bahrain Medical University, Manamah, Bahrain; l Research and Scientific Studies Unit, College of Nursing & Allied Health Sciences, Jazan University, Jazan, Saudi Arabia; m Medical Research Unit, School of Medicine, Universitas Syiah Kuala, Banda Aceh, Indonesia; n Tropical Disease Centre, School of Medicine, Universitas Syiah Kuala, Banda Aceh, Aceh, Indonesia; o Department of Microbiology, School of Medicine, Universitas Syiah Kuala, Banda Aceh, Aceh, Indonesia; p Semillero de Investigación en Zoonosis (SIZOO), Grupo de Investigación BIOECOS, Fundación Universitaria Autónoma de las Américas, Pereira, Risaralda, Colombia; q Public Health and Infection Research Group, Faculty of Health Sciences, Universidad Tecnologica de Pereira, Pereira, Colombia; r College of Horticulture and Forestry, Rani Lakshmi Bai Central Agricultural University, Jhansi, India; s Division of Pathology, ICAR-Indian Veterinary Research Institute, Bareilly, Uttar Pradesh, India; t Grupo De Investigacion Biomedicina, Faculty of Medicine, Fundacion Universitaria Autonoma de las Americas, Pereira, Colombia

**Keywords:** SARS-CoV-2, COVID-19, vaccine, immunotherapeutics, pandemic

## Abstract

The COVID-19 pandemic caused by SARS-CoV-2 has resulted in millions of cases and hundreds of thousands of deaths. Beyond there being no available antiviral therapy, stimulating protective immunity by vaccines is the best option for managing future infections. Development of a vaccine for a novel virus is a challenging effort that may take several years to accomplish. This mini-review summarizes the immunopathological responses to SARS-CoV-2 infection and discusses advances in the development of vaccines and immunotherapeutics for COVID-19.

## Introduction

The 2019 coronavirus disease (COVID-19) was first reported in late December 2019 in Wuhan; China has now become a global pandemic. The virus causing COVID-19 is the Severe acute respiratory coronavirus syndrome 2 (SARS-CoV-2) that belongs to the coronaviridae family of viruses. The characteristic feature of coronavirus is the presence of club-like extensions on the surface made of glycosylated trimers of S protein. The coronaviruses are roughly 80–120 nm in diameter.^1^ COVID-19 resulting from the new SARS-CoV-2 infection has now become a global health concern. The incubation time of the virus is about 2–10 days, and it is transmitted through aerosol from human-to-human and also through contaminated inanimate objects and hands.^2^ The virus can remain infective on the surfaces of objects for up to 9 days at room temperature. However, the viral survival declines with temperatures above 30°C. It can be efficiently inactivated by surface disinfection procedures with 62–71% ethanol, 0.5% hydrogen peroxide or 0.1% sodium hypochlorite within 1 minute.^2^

First described in China, SARS-CoV-2 has been reported in essentially all countries worldwide, with more than 15 million infected subjects and more than a half-million deaths. Owing to the global spread, WHO declared COVID-19 as a pandemic on 11 March 2020.^3^

Since the first report of the genomic sequence of the SARS-CoV-2 has come, researchers, clinicians, and pharmaceutical companies have devoted all their resources and research toward developing therapeutic modalities and vaccines for SARS-CoV-2.

Most of the data on antiviral therapy is based on the clinical and preclinical studies on other related viruses such as SARS-CoV, Middle East coronavirus respiratory syndrome (MERS-CoV) and non-coronavirus (Ebola). This narrative mini-review summarizes epidemiology, pathogenesis, immune responses, vaccine development issues, and immunotherapy for COVID-19 and provides an update on recent advances for vaccine and immunotherapy.

## Pathogenesis

The SARS-CoV-2 resembles the SARS-CoV in several aspects. Homology modeling revealed that both the viruses employ similar receptor-binding domains to attach to the host cells with subtle differences in particular amino acid residues.^4^ The coronaviruses have spike proteins that are glycoproteins and consist of two subunits: S1 and S2. The S1 and S2 proteins are the most important structural proteins of the virus. The spikes on the surface of the SARS-CoV-2 are homotrimers of S proteins that establish attachments with the host cell receptors.^5^ The structural and the non-structural proteins (nsps) in co-ordination carry out the CoV pathogenesis and decide the virulence.^6^

## The virus entry

Coronaviruses enter into the host cells by using the viral S protein.^7^ SARS-CoV-2 enters into the host cell by the interaction of its S protein with the host receptor “ACE2” present in most of the human cell types.^8^ The viral RNA is transferred into the host cell cytoplasm as soon as it enters the host cell. The viral genome translates its two polyproteins and structural proteins. These proteins enable the viral genome to replicate inside the host cell.^9^ The nascent viral glycoprotein envelope is processed in the endoplasmic reticulum or Golgi membrane. Then, the genomic RNA and the nucleocapsid proteins fuse to form the nucleocapsid. The newly formed viral particles then fuse with the vesicles in the intermediate reticulum-Golgi endoplasmic compartment (ERGIC) followed by the fusion of these virus-containing vesicles with the plasma membrane that leads to the virus release.^7^

## Antigen presentation

To date, there are no reported studies on the immune mechanism of SARS-CoV-2 infection. However, the studies on the immune mechanisms of the related viruses like SARS-CoV and MERS give much insight into the immune mechanism of the virus.^10^ Upon entry into the host, the virus presents its antigens to the antigen-presenting cells (APCs) of the host mediating the antiviral mechanism of the host immune system.

## Humoral and cellular immunity

Antigen presentation by the APCs results in the activation of the cell-mediated and the humoral immunity of the host governed by T cells and B cells, respectively. The antibody response (levels of IgM and IgG) to SARS-CoV follows a characteristic pattern.^11^ The IgM antibody levels reach undetectable levels by the end of 12th week of infection, but the IgG remains for more extended periods.^12^

SARS-CoV infection induces concomitant activation of T cell and B cell-mediated immune responses. Upon SARS-CoV infection, B cell responses are first observed against the nucleocapsid (N) protein followed by responses to S protein which is seen within 4–8 days after the onset of symptoms.^13,14^ Neutralizing antibody responses for the S protein begins by 2nd week. Many patients develop the neutralizing antibody responses by 3rd week.^15,16^ Since viral titers are observed to peak earlier for SARS-CoV-2 as compared to SARS-CoV, the antibody responses may also be elicited earlier.^17,18^ It has been observed that a subset of infected patients do not develop long-lasting antibody responses to SARS-CoV-2. However, it is not clear whether these patients are more susceptible for re-infection.^19,20^

It has been documented that the population of CD4 and CD8 T cells significantly falls in the patients infected with SARS-CoV-2.^21^ It was seen that the antibody-secreting cells (ASCs) in the blood of a SARS-CoV-2-infected patient increased from day 7 (1.48%) to a peak level on day 8 (6.91%). Similarly, the cT_FH_ cells increased from 1.98% on day 7 to 3.25% on day 8. The cT_FH_ cells peaked on 9th day (4.4.6%). This observation indicates that both humoral and cell-mediated immunity comes into play in response to SARS-CoV-2 infection.^22^

An accumulation of mononuclear cells suspected to be monocytes and T cells was observed in the lungs of a COVID-19 patient along with decreased systemic levels of hyperactive T cells.^21^ Lymphopenia and decreased levels of peripheral T cells indicate that the T cells are migrated toward the lungs from the systemic circulation to the site of infection (primarily lungs) to counteract the viral infection.^23–26^ Increased exhaustion and reduced functional diversity of T cells may be predictive of severe disease progression.^27^ It has been seen that the patients recovered from SARS-CoV developed coronavirus-specific memory T cells, seen up to 2 years after recovery.^28,29^ It is quite evident from these reports that T cell-mediated immunity plays an important role in controlling infection. However, several vaccine agents designed against SARS-CoV resulted in immunopathology due to TH2 cell-mediated infiltration of eosinophils.^30,31^ The vaccinated mice showed increased immunopathology than protection against SARS-CoV infection.^32^ Therefore, further extensive studies are needed to evaluate the protective versus damaging T cell responses, which is essential for designing vaccines for the coronavirus.^33^ However, it is very important to investigate whether T cell-mediated responses are solely responsible for infection control in humans. This will provide impetus to the vaccine development process.

## Cytokine storm

Acute respiratory distress syndrome (ARDS) is the most common pathology seen in patients infected with SARS-CoV-2, SARS-CoV, and MERS.^21,34^ A hyperinflammatory condition associated with hypercytokinaemia is often observed in COVID-19 patients.^34^ This hyperinflammatory condition is related to multiple organ failure.^35^ The cytokine surge seen in COVID-19 patients results due to increased levels of the proinflammatory cytokines such as IFN-a, IFN-g, IL-1b, IL-6, IL-12, IL-18, IL-33, TNF-a, TGFb and chemokines such as CCL2, CCL3, CCL5, CXCL8, CXCL9, CXCL10, among others, by the cells of immunity system.^34,36^ This cytokine storm may be followed by multiple organ failure and ARDS resulting in the death of the patients infected with SARS-COV-2 as seen in SARS-CoV and MERS-CoV infection.^21^

## Evasion of the host immune response

As the SARS-CoV-2 is very new to the researchers, not much data are available on the immunopathological mechanisms and the tricks of the novel virus to escape the host immune response. However, data from the studies on the previously known coronaviruses like SARS-CoV and MERS can be utilized to speculate the possible mechanisms this new SARS-CoV-2 virus may employ to deceive the host immune system. The pattern recognition receptors (PRRs) can identify the evolutionarily preserved microbial structures called pathogen-associated molecular patterns (PAMPs). As a defense mechanism against the host cells, these viruses (SARS-CoV and MERS-CoV) may develop modified membranes derived from host cell components such as developing double-membrane vesicles that lack or have altered pathogen-associated molecular patterns (PAMPs) hence unrecognizable by the host cell pattern recognition receptors (PRRs). This facilitates viral replication without their RNA being recognized by the host cell.^37^ Another mechanism seen in MERS infection can be thought of being utilized by the SARS-CoV-2 virus to escape the host immunity. It has been recognized with MERS infection that the expression of genes related to antigen presentation is downregulated upon infection, facilitating the virus evade the first checkpoint of host immunity.^38^ Research studies have evidenced the fact that nsp can suppress the innate immune response of the host.^6^

## Need for vaccine development

Although remdesivir is available in developed countries as a specific antiviral drug that has been shown to be effective in reducing symptoms and accelerating recovery from COVID-19 disease, it is not affordable in most parts of the world and is in limited supply, such that much of the world awaits an inexpensive and effective antiviral drug. The treatment for COVID-19 relies on the management of the symptoms focused on the symptomatic management of the patients which include controlling secondary infections by the administration of broad-spectrum antibiotics, ventilation, and fluid control.^11,34^

## COVID-19 vaccine development platforms and challenges in COVID-19 vaccine development

The public health threat of COVID-19 will remain until a potential and effective vaccine is developed.^39^ Several companies have taken the initiative of developing the vaccine against COVID-19 targeting the SARS-CoV2 virus continuously circulating in the human population.^40^

Various strategies such as live-attenuated virus, viral proteins, viral nucleic acid, virus-like particle, peptide, viral vector (replicating and non-replicating), and recombinant protein approaches are being used for vaccine development against SARS-CoV-2. These strategies have their own associated advantages and disadvantages.^5,41^ Viral antigen-based and nucleic acid-based vaccines are safer, but the immunogenic potential of these vaccines is less. The nucleic acid-based vaccines are the fastest to enter into phase 1 clinical trial, but to date, there is no nucleic acid-based vaccine licensed to be used in humans. Since safety is a concern with a live-attenuated virus, it is risky for the vulnerable older population.^41^ Nucleic acid-based platforms provide wider options for antigen manipulation. Therefore, vaccines based on nucleic acid approaches might be considered as one of the important approaches for the development of a vaccine for SARS-CoV2.^42^

Viral vector-based platforms offer advantages such as high level of protein expression, stability, and induction of strong immune response. Since vaccines based on recombinant proteins are already licensed for several other diseases, the existing large-scale production capacity can be utilized for the production of vaccines for SARS-CoV-2.^42^ It is possible that some platforms may be more appropriate for a specific group of population such as the aged, children, pregnant women or immunocompromised patients.

Since our knowledge about the immune responses to the vaccines is not entirely established, different strategies should be tried for designing a vaccine for COVID-19. Only advances and comprehensive research can answer this question about selecting the best approach for vaccine development for COVID-19. Distinctive challenges for SARS-CoV-2 vaccine development include clinical recruitment, defining a correlate of protection, and proving efficacy, especially when there is public pressure to release a vaccine for general use. Despite the development of novel platforms for vaccine development, there are several underlying challenges in SARS-CoV-2 vaccine development.

Although the viral S protein is a potential immunogen, it is essential to evaluate the optimal immune response elicited by the antigen. It is still a matter of debate whether the full S protein or only the receptor-binding domain is suitable for achieving optimal immune response.^33^ Previous experience with SARS and MERS vaccine candidates has raised concerns about the exacerbating lung disease, either directly or due to antibody-dependent enhancement (ADE). This adverse effect may be associated with Th2 response. Production of sub-optimal levels of antibody or antibody of low quality can result in the phenomenon of ADE that promotes the disease pathology. This warrants consideration of ADE in the evaluation of safety of the emerging candidate vaccines for SARS-CoV-2. Therefore, testing of the vaccine candidates for safety in suitable animal models and constant safety monitoring in clinical trials is quintessential. It is premature to define a good animal model for COVID-19.^33^

Another major challenge in SARS-CoV-2 vaccine development is establishment of correlates of protection. Experience with SARS and MERS vaccines may be utilized to establish correlates of protection. However, they are still not established. The duration of protection and whether a single dose of the vaccine is sufficient to elicit the required immune response is uncertain.^33^

Vaccine development is a long process and involves large sample sizes for conducting research; it may take years to establish a vaccine. Since it is associated with high costs and failure rates, the developers take extra precaution and follow a strict sequence of steps involving multiple rounds of data analysis and strict checks at manufacturing levels. Developing a vaccine in an outbreak scenario requires a new strategy of executing multiple steps in parallel even before confirmation of the outcome of another step.^33^

The other challenge is faced at the commercial production of clinical trial materials. For the novel platforms, large-scale production has never been done before. So, it will require large-scale production (identification, technology transfer, and manufacturing process) without the knowledge of the viability of the vaccine candidate. It is not certain whether these novel platforms are scalable and if it is possible to produce sufficient quantities using the existing capacity.^33^

Performing clinical trials during a pandemic is associated with the additional challenge of choosing trial sites as it is difficult to predict where and when outbreaks will occur. So, if multiple vaccines are ready, countries should not be crowded with multiple clinical trials. In a pandemic situation where the mortality rates are high, people may not consent for randomized controlled trials with placebo.^43^ One way to tackle this problem is utilizing a single-shared control group for testing several vaccines simultaneously. In this approach, more people will receive an active vaccine.^44^ Although this approach is advantageous, it is associated with statistical complexities. Developers may try to avoid direct comparative analysis of their vaccine candidates with others.

Finally, there will be a surge of demand for the vaccines globally. Hence, serological studies will be needed to establish which populations are at higher risks so that they can be prioritized for the vaccine allocation.^33^

## Advances in vaccine development for COVID-19

Several companies, research laboratories and universities are researching to come up with a vaccine for COVID-19. According to WHO, as of August 25, 2020, there are 31 vaccines in the clinical evaluation phase (Table 1) and 142 vaccines in the preclinical evaluation phase (according to the WHO draft, August 25, 2020) (Table 2).^45^ Out of the 31 candidate vaccines in clinical trial phase, 7 have reached phase 3, 3 have reached phase 2 clinical trials, and the rest are in phase 1 or phase1/2. Of the seven candidate vaccines in phase 3, ChAdOx1-S is a single dose intramuscular non-replicating viral vector vaccine expressing the SARS-CoV-2 spike protein. Other three are inactivated double dose intramuscular SARS-CoV-2 vaccines. LNP-encapsulated mRNA and 3 LNP-mRNAs are double dose intramuscular RNA vaccines. Ad26COVS1 is a double dose intramuscular non-replicating viral vector vaccine (Table 1). Three vaccine candidates listed in Table 1 are in phase 2 clinical trial. Adenovirus Type 5 Vector is a single dose intramuscular non-replicating viral vector vaccine. Adjuvanted recombinant protein (RBD-Dimer) is a double or triple dose intramuscular protein subunit vaccine. The other vaccine candidate in phase 2 clinical trial is a double dose intramuscular mRNA vaccine (Table 1).

**Table 1.** List of 31 candidate vaccines in different clinical trial phases.^45^***^.^***

						Clinical Stage			
COVID-19 Vaccine developer/manufacturer	Vaccine platform	Type of candidate vaccine	Number of doses	Timing of doses	Route of Administration	Phase 1	Phase 1/2	Phase 2	Phase 3
University of Oxford/AstraZeneca	Non-Replicating Viral Vector	ChAdOx1-S	1		IM		PACTR202006922165132 2020–001072-15	2020–001228-32	ISRCTN89951424 NCT04516746
Sinovac	Inactivated	Inactivated	2	0, 14 days	IM		NCT04383574 NCT04352608		NCT04456595 669/UN6.KEP/EC/2020
Wuhan Institute of Biological Products/Sinopharm	Inactivated	Inactivated	2	0,14 or 0,21 days	IM		ChiCTR2000031809		ChiCTR2000034780
Beijing Institute of Biological Products/Sinopharm	Inactivated	Inactivated	2	0,14 or 0,21 days	IM		ChiCTR2000032459		ChiCTR2000034780
Moderna/NIAID	RNA	LNP-encapsulated mRNA	2	0, 28 days	IM	NCT04283461 Interim Report		NCT04405076	NCT04470427
BioNTech/Fosun Pharma/Pfizer	RNA	3 LNP-mRNAs	2	0, 28 days	IM		2020–001038-36 ChiCTR2000034825 Study Report		NCT04368728
CanSino Biological Inc./Beijing Institute of Biotechnology	Non-Replicating Viral Vector	Adenovirus Type 5 Vector	1		IM	ChiCTR2000030906 Study Report		ChiCTR2000031781 Study Report	
Anhui Zhifei Longcom Biopharmaceutical/Institute of Microbiology, Chinese Academy of Sciences	Protein Subunit	Adjuvanted recombinant protein (RBD-Dimer)	2 or 3	0, 28 or 0, 28, 56 days	IM	NCT04445194		NCT04466085	
Curevac	RNA	mRNA	2	0, 28 days	IM	NCT04449276		NCT04515147	
Institute of Medical Biology, Chinese Academy of Medical Sciences	Inactivated	Inactivated	2	0, 28 days	IM	NCT04412538	NCT04470609		
Inovio Pharmaceuticals/International Vaccine Institute	DNA	DNA plasmid vaccine with electroporation	2	0, 28 days	ID		NCT04447781 NCT04336410		
Osaka University/AnGes/Takara Bio	DNA	DNA plasmid vaccine + Adjuvant	2	0, 14 days	IM		NCT04463472		
Cadila Healthcare Limited	DNA	DNA plasmid vaccine	3	0, 28, 56 days	ID		CTRI/2020/07/026352		
Genexine Consortium	DNA	DNA Vaccine (GX-19)	2	0, 28 days	IM		NCT04445389		
Bharat Biotech	Inactivated	Whole-Virion Inactivated	2	0, 14 days	IM		NCT04471519		
Janssen Pharmaceutical Companies	Non-Replicating Viral Vector	Ad26COVS1	2	0, 56 days	IM		NCT04436276		NCT04505722 (not yet recruiting)
Novavax	Protein Subunit	Full length recombinant SARS CoV- 2 glycoprotein nanoparticle vaccine adjuvanted with Matrix M	2	0, 21 days	IM		NCT04368988		
Kentucky Bioprocessing, Inc	Protein Subunit	RBD-based	2	0, 21 days	IM		NCT04473690		
Arcturus/Duke-NUS	RNA	mRNA			IM		NCT04480957		
Gamaleya Research Institute	Non-Replicating Viral Vector	Adeno-based	1		IM	NCT04436471 NCT04437875			
ReiThera/LEUKOCARE/Univercells	Non-Replicating Viral Vector	Replication defective Simian Adenovirus (GRAd) encoding S	1		IM	2020–002835-31			
Clover Biopharmaceuticals Inc./GSK/Dynavax	Protein Subunit	Native like Trimeric subunit Spike Protein vaccine	2	0, 21 days	IM	NCT04405908			
Vaxine Pty Ltd/Medytox	Protein Subunit	Recombinant spike protein with Advax™ adjuvant	1		IM	NCT04453852			
University of Queensland/CSL/Seqirus	Protein Subunit	Molecular clamp stabilized Spike protein with MF59 adjuvant	2	0, 28 days	IM	ACTRN12620000674932p			
Medigen Vaccine Biologics Corporation/NIAID/Dynavax	Protein Subunit	S-2P protein + CpG 1018	2	0, 28 days	IM	NCT04487210			
Instituto Finlay de Vacunas, Cuba	Protein Subunit	RBD + Adjuvant	2	0, 28 days	IM	IFV/COR/04			
Institute Pasteur/Themis/Univ. of Pittsburg CVR/Merck Sharp & Dohme	Replicating Viral Vector	Measles-vector based	1 or 2	0, 28 days	IM	NCT04497298			
Imperial College London	RNA	LNP-nCoVsaRNA	2		IM	ISRCTN17072692			
People’s Liberation Army (PLA) Academy of Military Sciences/Walvax Biotech.	RNA	mRNA	2	0, 14 or 0, 28 days	IM	ChiCTR2000034112			
Medicago Inc.	VLP	Plant-derived VLP adjuvanted with GSK or Dynavax adjs.	2	0, 21 days	IM	NCT04450004			
FBRI SRC VB VECTOR, Rospotrebnadzor, Koltsovo	Protein Subunit	Peptide	2	0, 21 days	IM	TBD			

Abbreviations: IM = Intra muscular’

**Table 2.** List of 142 candidate vaccines in preclinical evaluation phase.^45.^

Platform	Type of candidate vaccine	Developer	Same platform for non-Coronavirus candidates
DNA	DNA, engineered vaccine inserts compatible with multiple delivery systems	DIOSynVax Ltd / University of Cambridge	
DNA	DNA vaccine	Ege University	
DNA	DNA plasmid vaccine RBD&N	Scancell/University of Nottingham/ Nottingham Trent University	
DNA	DNA plasmid vaccine S,S1,S2,RBD &N	National Research Centre, Egypt	
DNA	DNA with electroporation	Karolinska Institute / Cobra Biologics (OPENCORONA Project)	
DNA	DNA with electroporation	Chula Vaccine Research Center	
DNA	DNA	Takis/Applied DNA Sciences/Evvivax	
DNA	Plasmid DNA, Needle-Free Delivery	Immunomic Therapeutics, Inc./EpiVax, Inc./PharmaJet	SARS
DNA	DNA vaccine	BioNet Asia	
DNA	msDNA vaccine	Mediphage Bioceuticals/University of Waterloo	
DNA	DNA vaccine	Entos Pharmaceuticals	
DNA	bacTRL-Spike	Symvivo	
Inactivated	Inactivated + alum	KM Biologics	JE, Zika
Inactivated	Inactivated	Selcuk University	
Inactivated	Inactivated	Erciyes University	
Inactivated	Inactivated whole virus	National Research Centre, Egypt	
Inactivated	Inactivated	Beijing Minhai Biotechnology Co., Ltd.	
Inactivated	TBD	Osaka University/ BIKEN/ NIBIOHN	
Inactivated	Inactivated + CpG 1018	Sinovac/Dynavax	
Inactivated	Inactivated + CpG 1018	Valneva/Dynavax	
Inactivated	Inactivated	Research Institute for Biological Safety Problems, Rep of Kazakhstan	
Live Attenuated Virus	Codon deoptimized live attenuated vaccines	Mehmet Ali Aydinlar University / Acıbadem Labmed Health Services A.S.	
Live Attenuated Virus	Codon deoptimized live attenuated vaccines	Codagenix/Serum Institute of India	HAV, InfA, ZIKV, FMD, SIV, RSV, DENV
Live Attenuated Virus	Codon deoptimized live attenuated vaccines	Indian Immunologicals Ltd/Griffith University	
Non-Replicating Viral Vector	Sendai virus vector	ID Pharma	
Non-Replicating Viral Vector	Adenovirus-based	Ankara University	
Non-Replicating Viral Vector	Adeno-associated virus vector (AAVCOVID)	Massachusetts Eye and Ear/Massachusetts General Hospital/AveXis	
Non-Replicating Viral Vector	MVA encoded VLP	GeoVax/BravoVax	LASV, EBOV, MARV, HIV
Non-replicating viral vector	MVA-S encoded	DZIF – German Center for Infection Research/IDT Biologika GmbH	Many
Non-replicating viral vector	MVA-S	IDIBAPS-Hospital Clinic, Spain	
Non-Replicating Viral Vector	adenovirus-based NasoVAX expressing SARS2-CoV spike protein	Altimmune	influenza
Non-Replicating Viral Vector	Adeno5-based	Erciyes University	
Non-Replicating Viral Vector	2nd Gen E2b- Ad5 Spike, RBD, Nucleocapsid Subcutaneous&Oral	ImmunityBio, Inc. & NantKwest, Inc.	flu, Chik, Zika, EBOV, LASV, HIV/SIV,Cancer
Non-Replicating Viral Vector	Ad5 S (GREVAX™ platform)	Greffex	MERS
Non-Replicating Viral Vector	Oral Ad5 S	Stabilitech Biopharma Ltd	Zika, VZV, HSV-2 and Norovirus
Non-Replicating Viral Vector	adenovirus-based + HLA-matched peptides	Valo Therapeutics Ltd	
Non-Replicating Viral Vector	Oral Vaccine platform	Vaxart	InfA, CHIKV, LASV, NORV; EBOV, RVF, HBV, VEE
Non-Replicating Viral Vector	MVA expressing structural proteins	Centro Nacional Biotecnología (CNB-CSIC), Spain	Multiple candidates
Non-Replicating Viral Vector	Dendritic cell-based vaccine	University of Manitoba	
Non-Replicating Viral Vector	parainfluenza virus 5 (PIV5)-based vaccine expressing the spike protein	University of Georgia/University of Iowa	MERS
Non-Replicating Viral Vector	Recombinant deactivated rabies virus containing S1	Bharat Biotech/Thomas Jefferson University	HeV, NiV, EBOV, LASSA, CCHFV, MERS
Non-Replicating Viral Vector	Influenza A H1N1 vector	National Research Centre, Egypt	
Non-Replicating Viral Vector	Inactivated Flu-based SARS-CoV2 vaccine + Adjuvant	National Center for Genetic Engineering and Biotechnology (BIOTEC) /GPO, Thailand	
Protein Subunit	RBD protein (baculovirus production) + FAR- Squalene adjuvant	Farmacológicos Veterinarios SAC (FARVET SAC) / Universidad Peruana Cayetano Heredia (UPCH)	
Protein Subunit	Protein Subunit	Research Institute for Biological Safety Problems, Rep of Kazakhstan	
Protein Subunit	RBD-protein	Mynvax	
Protein Subunit	Recombinant S protein	Izmir Biomedicine and Genome Center	
Protein Subunit	Peptide + novel adjuvant	Bogazici University	
Protein Subunit	S subunit intranasal liposomal formulation with GLA/3M052 adjs.	University of Virginia	
Protein Subunit	S-Protein (Subunit) + Adjuvant, E coli based Expression	Helix Biogen Consult, Ogbomoso & Trinity Immonoefficient Laboratory, Ogbomoso, Oyo State, Nigeria.	
Protein Subunit	Protein Subunit S,N,M&S1 protein	National Research Centre, Egypt	
Protein Subunit	Protein Subunit	University of San Martin and CONICET, Argentina	
Protein Subunit	RBD protein fused with Fc of IgG + Adj.	Chulalongkorn University/GPO, Thailand	
Protein Subunit	Capsid-like Particle	AdaptVac (PREVENT-nCoV consortium)	
Protein Subunit	Drosophila S2 insect cell expression system VLPs	ExpreS2ion	
Protein Subunit	Peptide antigens formulated in LNP	IMV Inc	
Protein Subunit	S protein	WRAIR/USAMRIID	
Protein Subunit	S protein +Adjuvant	National Institute of Infectious Disease, Japan/Shionogi/UMN Pharma	Influenza
Protein Subunit	VLP-recombinant protein + Adjuvant	Osaka University/ BIKEN/ National Institutes of Biomedical Innovation, Japan	
Protein Subunit	microneedle arrays S1 subunit	Univ. of Pittsburgh	MERS
Protein Subunit	Peptide	Vaxil Bio	
Protein Subunit	Adjuvanted protein subunit (RBD)	Biological E Ltd	
Protein Subunit	Peptide	Flow Pharma Inc	Ebola, Marburg, HIV, Zika, Influenza, HPV therapeutic vaccine, BreastCA vaccine
Protein Subunit	S protein	AJ Vaccines	
Protein Subunit	Ii-Key peptide	Generex/EpiVax	Influenza, HIV, SARS-CoV
Protein Subunit	S protein	EpiVax/Univ. of Georgia	H7N9
Protein Subunit	Protein Subunit EPV-CoV-19	EpiVax	
Protein Subunit	S protein (baculovirus production)	Sanofi Pasteur/GSK	Influenza, SARS-CoV
Protein Subunit	gp-96 backbone	Heat Biologics/Univ. Of Miami	NSCLC, HIV, malaria, Zika
Protein Subunit	Subunit vaccine	FBRI SRC VB VECTOR, Rospotrebnadzor, Koltsovo	
Protein Subunit	S1 or RBD protein	Baylor College of Medicine	SARS
Protein Subunit	Subunit protein, plant produced	iBio/CC-Pharming	
Protein Subunit	Recombinant protein, nanoparticles (based on S-protein and other epitopes)	Saint-Petersburg scientific research institute of vaccines and serums	
Protein Subunit	COVID-19 XWG-03 truncated S (spike) proteins	Innovax/Xiamen Univ./GSK	HPV
Protein Subunit	Adjuvanted microsphere peptide	VIDO-InterVac, University of Saskatchewan	
Protein Subunit	Synthetic Long Peptide Vaccine candidate for S and M proteins	OncoGen	
Protein Subunit	Oral E. coli-based protein expression system of S and N proteins	MIGAL Galilee Research Institute	
Protein Subunit	Nanoparticle vaccine	LakePharma, Inc.	
Protein Subunit	Plant-based subunit (RBD-Fc + Adjuvant)	Baiya Phytopharm/ Chula Vaccine Research Center	
Protein Subunit	OMV-based vaccine	Quadram Institute Biosciences	Flu A, plague
Protein Subunit	OMV-based vaccine	BiOMViS Srl/Univ. of Trento	
Protein subunit	structurally modified spherical particles of the tobacco mosaic virus (TMV)	Lomonosov Moscow State University	rubella, rotavirus
Protein Subunit	Spike-based	University of Alberta	Hepatitis C
Protein Subunit	Recombinant S1-Fc fusion protein	AnyGo Technology	
Protein Subunit	Recombinant protein	Yisheng Biopharma	
Protein Subunit	Recombinant S protein in IC-BEVS	Vabiotech	
Protein Subunit	Orally delivered, heat stable subunit	Applied Biotechnology Institute, Inc.	
Protein Subunit	Peptides derived from Spike protein	Axon Neuroscience SE	
Protein Subunit	Protein Subunit	MOGAM Institute for Biomedical Research, GC Pharma	
Protein Subunit	RBD-based	Neovii/Tel Aviv University	
Protein Subunit	Outer Membrane Vesicle (OMV)-subunit	Intravacc/Epivax	
Protein Subunit	Outer Membrane Vesicle(OMV)-peptide	Intravacc/Epivax	
Protein Subunit	Spike-based (epitope screening)	ImmunoPrecise/LiteVax BV	
Replicating Bacteria Vector	Oral Salmonella enteritidis (3934Vac) based protein expression system of RBD	Farmacológicos Veterinarios SAC (FARVET SAC) / Universidad Peruana Cayetano Heredia (UPCH)	
Replicating Viral Vector	YF17D Vector	KU Leuven	
Replicating Viral Vector	Measles Vector	Cadila Healthcare Limited	
Replicating Viral Vector	Measles Vector	FBRI SRC VB VECTOR, Rospotrebnadzor, Koltsovo	
Replicating Viral Vector	Measles Virus (S, N targets)	DZIF – German Center for Infection Research/CanVirex AG	Zika, H7N9, CHIKV
Replicating Viral Vector	Horsepox vector expressing S protein	Tonix Pharma/Southern Research	Smallpox, monkeypox
Replicating Viral Vector	Live viral vectored vaccine based on attenuated influenza virus backbone (intranasal)	BiOCAD and IEM	Influenza
Replicating Viral Vector	Recombinant vaccine based on Influenza A virus, for the prevention of COVID-19 (intranasal)	FBRI SRC VB VECTOR, Rospotrebnadzor, Koltsovo	Influenza
Replicating Viral Vector	Attenuated Influenza expressing an antigenic portion of the Spike protein	Fundação Oswaldo Cruz and Instituto Buntantan	Influenza
Replicating Viral Vector	Influenza vector expressing RBD	University of Hong Kong	
Replicating Viral Vector	Replication-competent VSV chimeric virus technology (VSVΔG) delivering the SARS-CoV- 2 Spike (S) glycoprotein.	IAVI/Merck	Ebola, Marburg, Lassa
Replicating Viral Vector	Replicating VSV vector-based DC-targeting	University of Manitoba	
Replicating Viral Vector	VSV-S	University of Western Ontario	HIV, MERS
Replicating Viral Vector	VSV-S	Aurobindo	
Replicating Viral Vector	VSV vector	FBRI SRC VB VECTOR, Rospotrebnadzor, Koltsovo	
Replicating Viral Vector	VSV-S	Israel Institute for Biological Research/Weizmann Institute of Science	
Replicating Viral Vector	M2-deficient single replication (M2SR) influenza vector	UW–Madison/FluGen/Bharat Biotech	influenza
Replicating Viral Vector	Newcastle disease virus vector (NDV-SARS- CoV-2/Spike)	Intravacc/ Wageningen Bioveterinary Research/Utrecht Univ.	
Replicating Viral Vector	Avian paramyxovirus vector (APMV)	The Lancaster University, UK	
RNA	Self-amplifying RNA	Gennova	
RNA	mRNA	Selcuk University	
RNA	LNP-mRNA	Translate Bio/Sanofi Pasteur	
RNA	LNP-mRNA	CanSino Biologics/Precision NanoSystems	
RNA	LNP-encapsulated mRNA cocktail encoding VLP	Fudan University/ Shanghai JiaoTong University/RNACure Biopharma	
RNA	LNP-encapsulated mRNA encoding RBD	Fudan University/ Shanghai JiaoTong University/RNACure Biopharma	
RNA	Replicating Defective SARS-CoV-2 derived RNAs	Centro Nacional Biotecnología (CNB-CSIC), Spain	
RNA	LNP-encapsulated mRNA	University of Tokyo/ Daiichi-Sankyo	MERS
RNA	Liposome-encapsulated mRNA	BIOCAD	
RNA	Several mRNA candidates	RNAimmune, Inc.	
RNA	mRNA	FBRI SRC VB VECTOR, Rospotrebnadzor, Koltsovo	
RNA	mRNA	China CDC/Tongji University/Stermina	
RNA	LNP-mRNA	Chula Vaccine Research Center/University of Pennsylvania	
RNA	mRNA in an intranasal delivery system	eTheRNA	
RNA	mRNA	Greenlight Biosciences	
RNA	mRNA	IDIBAPS-Hospital Clinic, Spain	
VLP	VLP	Bezmialem Vakif University	
VLP	VLP	Middle East Technical University	
VLP	Enveloped Virus-Like Particle (eVLP)	VBI Vaccines Inc.	CMV, GBM, Zika
VLP	S protein integrated in HIV VLPs	IrsiCaixa AIDS Research/IRTA-CReSA/Barcelona Supercomputing Centre/Grifols	
VLP	VLP + Adjuvant	Mahidol University/ The Government Pharmaceutical Organization (GPO)/Siriraj Hospital	
VLP	Virus-like particles, lentivirus and baculovirus vehicles	Navarrabiomed, Oncoimmunology group	
VLP	Virus-like particle, based on RBD displayed on virus-like particles	Saiba GmbH	
VLP	ADDomerTM multiepitope display	Imophoron Ltd and Bristol University’s Max Planck Centre	
VLP	Unknown	Doherty Institute	
VLP	VLP	OSIVAX	
VLP	eVLP	ARTES Biotechnology	malaria
VLP	VLPs peptides/whole virus	Univ. of Sao Paulo	

## After the vaccine is developed

It is not the only challenge to come up with an effective vaccine for COVID-19 in a short period but the most significant problem would be getting enough doses of the vaccine to be supplied to the countries globally. There is a risk that the more affluent countries can monopolize on the supply of COVID-19 vaccines globally in a similar way that happened with the flu pandemic. Therefore, along with focusing on the vaccine development, we should focus on containing the spread of the virus. Economically weak countries as in Africa would face problems in accessing vaccines, as happened with anti-HIV drugs. Due to the high rates of HIV drugs, several poor people died in Africa not being able to afford it. Therefore, there should be a fair distribution of the vaccines if any company succeeds in the race of developing a vaccine for COVID-19.

## Convalescent plasma therapy

Convalescent plasma therapy is an old concept of separating serum from the blood of a patient who has recovered from infection and injecting it to another infected patient. The convalescent plasma contains the antibodies for the infectious pathogen which neutralizes the pathogen in the new recipient patient. This therapy can be useful in treating COVID-19 patients.

The evidence coming from studies that reported the use of convalescent plasma therapy in treating past coronavirus infections such as SARS and MERS compelled the researchers to apply this therapy on COVID-19 patients.^46–49^ Recent studies have highlighted the beneficial effects of convalescent plasma therapy in critically ill COVID-19 patients.^50^ Among the five critically ill COVID-19 patients who were administered convalescent plasma, three patients were discharged upon recovery, and two patients are in the incubation period of 37 days.^50^ This treatment modality is associated with some disadvantages. Convalescent plasma therapy increases the risk of serum related disease and antibody-dependent enhancement of infection. There is always the risk of transmission of other infectious diseases through the serum and the additional risk associated with convalescent plasma therapy is the chances of developing infection from another viral strain due to antibodies against one form of coronavirus.^51^ All published studies on clinical trials with convalescent plasma did not include a negative-control group needed to judge the efficacy of the intervention. Therefore, the need of the hour is the identification of the human monoclonal antibody for a common antigenic determinant/epitope of SARS CoV-2 to prevent COVID-19.

## Monoclonal antibody therapy

Human monoclonal antibodies such as 80 R, m396, and S230.15 specific for the S1 domain of the SARS CoV have been reported to be effective in neutralizing the SARS-CoV infections by inhibiting their binding to the ACE receptors on the host cells.^52^ Another study reported that the monoclonal antibody CR3014 reduced the rate of replication of SARS-CoV genome and inhibited viral shedding, thus wholly prevented the virus-induced lung pathology. This antibody also works on the principle of inhibiting the binding of SARS-CoV by reducing the affinity of the S1 domain of the virus for the ACE receptor on the host cells.^53^

Since the receptor-binding domain of SARS-CoV-2 differs significantly from the SARS-CoV virus, the monoclonal antibodies (as m396, CR3014) targeting the S1 domain of SARS-CoV may not be effective in neutralizing the novel SARS-CoV-2. A recent study highlighted that the human monoclonal antibody CR3022 completely neutralizes both SARS-CoV and SARS-CoV-2. Therefore, these monoclonal antibodies can be considered for use in the prevention and treatment of COVID-19.^54^ Another study reported that the human monoclonal antibody 47D11 neutralizes SARS-CoV-2 by binding to the conserved sequence on the receptor-binding domain of the S1B protein. These antibodies can slow down the viral infection and can impart immunity in the uninfected persons.^55^ The receptor-binding domain is the best target to develop monoclonal antibody treatment to manage or prevent SARS-CoV-2 infections.

Takeda Pharmaceutical Company based in Japan is in the process of preparing a monoclonal antibody mixture, TAK-888 from the serum of recovered COVID-19 patients to come up with a new treatment strategy for COVID-19. Another pharmaceutical company, Vir Pharmaceuticals, USA, is testing antibodies isolated from recovered SARS patients to neutralize SARS-CoV-2. This company has also collaborated with a China-based company, WuXi Biologics, to develop a serum-based therapy to tackle SARS-CoV-2 infection in critically ill patients.^56^

A limitation to the use of convalescent plasma and MAbs is that they might benefit hospitalized patients but will not be generally useful for the population.

## Conclusion

Several companies have initiated the development of antiviral and vaccines for COVID-19. Different approaches have been undertaken to develop an effective vaccine for COVID-19 such as attenuated virus, viral proteins, viral nucleic acid, virus-like particle, peptide, viral vector (replicating and non-replicating), and recombinant proteins. However, the most significant challenge post-vaccine development will be the fair distribution of the vaccines globally. Convalescent plasma therapy and monoclonal antibody therapy are also being tested and can be the potential therapeutic modality for the management and prevention of COVID-19. However, they might benefit only the hospitalized patients and will not be generally useful for the population.
